# The Many Faces of Mitochondrial Dysfunction in Depression: From Pathology to Treatment

**DOI:** 10.3389/fphar.2019.00995

**Published:** 2019-09-10

**Authors:** Giuseppe Caruso, Cristina Benatti, Joan M.C. Blom, Filippo Caraci, Fabio Tascedda

**Affiliations:** ^1^Oasi Research Institute, IRCCS, Troina, Italy; ^2^Department of Life Sciences, University of Modena and Reggio Emilia, Modena, Italy; ^3^Center for Neuroscience and Neurotechnology, University of Modena and Reggio Emilia, Modena, Italy; ^4^Department of Education and Human Sciences, University of Modena and Reggio Emilia, Modena, Italy; ^5^Department of Drug Sciences, University of Catania, Catania, Italy

**Keywords:** mitochondrion, depression, energetic metabolism, antidepressants, antioxidants

## Introduction

The last years of neurobiological research have transformed the way we consider mental illnesses. We have gone from a deterministic genetic view to a broader vision that includes the involvement of non-cerebral systems. This is especially true for major depression (MD). Historically, MD has been perceived as a multifactorial disorder correlated to various neurobiological changes like neurotransmitter deficits, endocrine disturbances, impaired plasticity, and neural adaptation (Benatti et al., 2016). Indeed, the development and progression of depressive disorders has been conceived as the disruption of body allostasis, defined as the process of achieving stability of physiological and mental processes through dynamic change (Wang et al., 2019). The main player in the “allostatic game” is the brain, an organ designed to integrate signals from the periphery that anticipate fluctuations, changes, and needs and coordinates allostatic mediators in order to develop successful coping mechanisms that ultimately lead to an adaptative strategy and resilience (de Kloet et al., 2005).

The establishment and maintenance of these mechanisms requires large amounts of energy from the organism. Without energy, or in a partial lack of energy, the biological mechanisms necessary to respond appropriately to stimuli may not occur or be established incorrectly or abnormally.

Human and animal studies suggest an intriguing link between our body’s ability to produce energy and the brain’s ability to correctly perform the complex cellular and molecular processes involved in allostatic processes.

In eukaryotic cells, mitochondria are the powerhouse that produces and distributes energy to all other components. Functional or quantitative alterations of the ability of mitochondria to adequately supply energy can have important repercussions primarily on cellular processes and cascades of serial events (Herst et al., 2017) as well as on the correct functioning of the organism including mechanisms of brain plasticity, mood, and behavior in general (Allen et al., 2018). In this framework, it is particularly intriguing to think of the mitochondria as an active regulator of many of the biological phenomena involved in depression and in the efficacy of or resistance to the most widely used pharmacological treatments.

Once the energetic equilibrium is compromised, the body becomes more “vulnerable.” This is especially true for stress-related disorders, such as depression. In fact, depression is often associated with energetic imbalance leading to profound effects on the disease (Zuccoli et al., 2017). The driving questions then are as follows: What happens to the brain in the presence of an energetic imbalance? Does depression or depression-related symptoms impact mitochondrial energetic efficiency? Is antidepressant efficacy mediated by mitochondrial functionality?

## Mitochondria and Depressive Disorders

**Figure 1:**
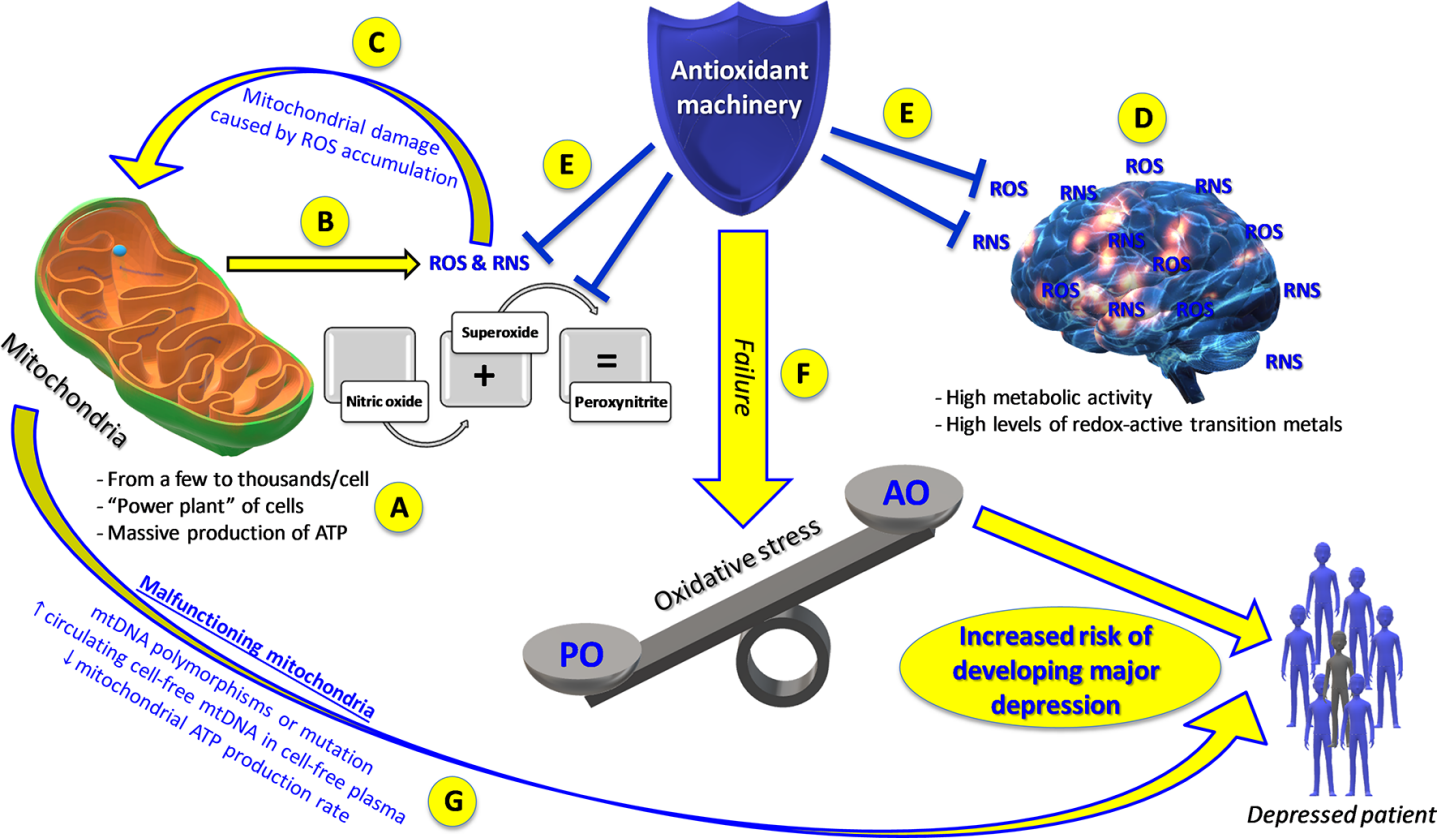
Mitochondrial dysfunction and oxidative stress: the “dangerous cocktail” increasing the risk of developing or triggering depression. **(A)** Mitochondrial physiology. **(B)** Brain mitochondria produce high quantities of reactive oxygen species (ROS) and reactive nitrogen species (RNS) along with adenosine triphosphate (ATP), increasing their own vulnerability **(C)** and that of the brain **(D)** to oxidative damage. When the ability of the antioxidant machinery to balance ROS/RNS production **(E)** fails, oxidative stress develops **(F)**. Both oxidative stress and malfunctioning mitochondria **(G)** represent two risk factors for the development of major depression. AO, antioxidants; PO, pro-oxidants.

To answer these questions, we need to (1) evaluate the effects of mitochondrial functions on depression or on some aspects of depression and (2) consider the possibility that the pathology itself or some of its neuroendocrine aspects modify mitochondrial function. Each of our cells contains a variable number (from a few to thousands) of small organelles called mitochondria (Pizzorno, 2014). These subcellular structures represent the “power plant” of cells, being responsible for a massive production of adenosine triphosphate (ATP), an indispensable molecule for life (Johannsen and Ravussin, 2009) (**Figure 1**). The activity of mitochondria is even more important for the brain, an organ that uses a huge quantity of ATP but that is not able to store large amounts of energy reserves (e.g. neurons do not store glucose) (Allen et al., 2018). Therefore, the integrity of mitochondrial activity is key to the continuous energy supply to the brain. Increasing evidence implicates mitochondrial dysfunction as a key player in the development of neuropsychiatric disorders, such as depression (Manji et al., 2012). In particular, deficient mitochondrial function is involved in impaired neuronal communication and cellular resilience, which in turn have been hypothesized to lead to mood disorders and psychotic disorders (Quiroz et al., 2008). Preclinical studies show that exposure to chronic mild stress, a well-established animal model of depression, induces depressive-like symptoms in mice and is accompanied by reduced mitochondrial respiratory rates and a dissipated mitochondrial membrane potential in the hippocampus, cortex, and hypothalamus (Gong et al., 2011). These observations support the hypothesis that mood disorders could be associated with an abnormal cerebral energy metabolism and, thus, strengthen the supposition that depression is caused by an impairment in energy in the brain due to mitochondrial genetic vulnerability [A3243G mutation, mitochondrial transfer RNALeu(UUR)] and environmental mechanisms (Onishi et al., 1997). Inflammation and oxidative stress exert a central role in the pathogenesis of MD (Joseph et al., 2005). Recently, Kageyama et al. (2018) showed a correlation between circulating mitochondrial DNA (mtDNA) and inflammation, measured as plasma levels of four cytokines [granulocyte macrophage colony-stimulating factor (GM-CSF), interleukin (IL)-2, IL-4, and IL-6], in MD patients. Patients with MD displayed heightened levels of pro-inflammatory cytokines, such as IL-6, IL-8, and tumor necrosis factor-α (TNF-α), while reduced levels of anti-inflammatory cytokines, such as transforming growth factor-β1 (TGF-β1), along with an increase in TNF-α levels were observed in the plasma of depressed patients (Caraci et al., 2018), which may significantly contribute to treatment resistance in MD. Therefore, a strong neurobiological link between inflammation, oxidative stress, and treatment resistance in MD has been hypothesized. Also, a poorer response to antidepressant treatment was related to higher baseline levels of the major oxidative stress marker F2-isoprostane, which in turn was associated with changes in oxidative (8-OHdG) and inflammatory (IL-6) markers (Lindqvist et al., 2017). Furthermore, patients with MD displayed higher levels of circulating cell-free mitochondrial DNA compared to healthy controls, while mtDNA copy number, reflecting intracellular mtDNA content and bioenergetics, did not differ between the groups (Lindqvist et al., 2018).

Levels of ATP are generally lower in the brain tissue of depressed patients than in healthy subjects, while in the muscle tissue of patients with MD, an enhanced ATP production rate and mtDNA were observed as compared to healthy controls (Moretti et al., 2003). Moreover, some studies suggest that the pharmacodynamics of different classes of antidepressant drugs includes a specific and selective influence on mitochondrial energy production mechanisms. As a result, these effects may play specific roles in the different cellular compartments, which is of particular significance when considering presynaptic and postsynaptic compartments (Villa et al., 2016; Villa et al., 2017).

In addition, a recent study expanded “the allostatic load model of chronic stress,” focusing on glucocorticoid dysregulation, introducing the concept of “mitochondrial allostatic load” (Picard et al., 2014), characterized by mitochondrial fragmentation, reactive oxygen species (ROS) production, mtDNA damage, an early event mediating the relationship between primary mediators of chronic stress (e.g. increased levels of cortisol, catecholamine, and blood glucose), and disease pathways in MD.

When present at low levels, ROS and reactive nitrogen species (RNS) represent a fundamental component of living organisms and are implicated in many physiological processes. The ability of the antioxidant machinery to keep the levels of ROS and RNS low, without ever reaching their complete elimination, is therefore extremely important (Cheignon et al., 2018). Among the producers of different reactive species, mitochondria are the most representative source (Cadenas and Davies, 2000). However, the brain, due to the presence of high levels of redox-active transition metals along with their high metabolic activity, is one of the most vulnerable organs to damage of ROS/RNS (Garbarino et al., 2015). Zhu et al. estimated that a resting cortical neuron consumes around five billion ATP molecules per second (Zhu et al., 2012). Inevitably, brain mitochondria produce high quantities of ROS and RNS together with ATP, making the brain increasingly vulnerable to oxidative damage. When the balance between pro-oxidants and antioxidants fails and pro-oxidants are in excess, a phenomenon called “oxidative stress” occurs (Birben et al., 2012). Two of the most-cited species, nitric oxide (NO) and superoxide anion are part of the natural energy metabolism of the cell while also being crucially implicated in oxidative stress (de Campos et al., 2015). The diffusion-limited reaction between NO and superoxide anion leads to the formation of peroxynitrite, the reactive species responsible for the damage of important biological macromolecules (Beckman and Crow, 1993). Currently, the hypothesis that all the above-mentioned species, and oxidative stress in general, play a fundamental role in depression is well accepted (Michel et al., 2012).

Preclinical and clinical studies together with various meta-analysis and systematic reviews strongly underline the role played by mitochondrial ROS/RNS metabolism and oxidative stress in depression (Liu et al., 2015) (**Figure 1**). Lipid peroxidation due to ROS generation is particularly relevant as the brain is rich in polyunsaturated lipids (Patel, 2016). Levels of malondialdehyde (MDA), a well-known marker of oxidative stress involved in lipid peroxidation, are increased, while those of antioxidants are decreased in depressed patients compared to healthy age- and sex-matched controls (Bajpai et al., 2014); levels of MDA were significantly higher, and those of ascorbic acid and superoxide dismutase (SOD) significantly lower in the serum of depressed patients compared to those of controls. Moreover, in depressed patients, an association has been observed between plasma MDA levels (higher than in healthy subjects) and severity of depressive symptoms. Further evidence of oxidative stress and antioxidant imbalance in depressed subjects as compared to healthy volunteers was provided by altered plasma levels of MDA, NO metabolites, antioxidant activity (SOD, vitamins E and C, and uric acid), and total antioxidant capacity. Also, the activity of several oxidative stress-related enzymatic systems has been linked to the pathogenesis of depression (Schiavone et al., 2013). For example, Ibi et al. (2017) found that mice deficient in NADPH oxidase 1 (Nox1^−/Y^), which displayed a depressive phenotype, showed the involvement of NOX1, as well as biochemical and structural changes. Specifically, depressive-like behaviors in mice were regulated by the NOX1 enzyme through redox modification of *N*-methyl-d-aspartate (NMDA) receptor 1. Inducible nitric oxide synthase (iNOS) and cyclooxygenase-2 (COX-2) represent two additional examples of enzymes whose activity has been linked to depression. A selective iNOS inhibitor (aminoguanidine), in streptozotocin-treated mice ameliorated cognitive deficits and depression and reduced the activity of iNOS (Zhou et al., 2017). Likewise, the COX-2 inhibitor celecoxib has therapeutic effects in patients diagnosed with MD suffering from an acute episode and has shown the same effects in animal models of depression as well (Muller and Schwarz, 2008). As mentioned above, patients with MD are characterized by a lowered total antioxidant state and by enhanced mitochondria-related oxidative stress, which is the reason why antioxidant supplementation is increasingly considered as a candidate treatment for depression (**Figure 1**). Converging evidence from studies employing both animal models of depression and human subjects provides a good perspective for the use of antioxidants in combination with antidepressants. Patients with depression had significantly lower levels of blood serum levels of vitamins A, C, and E in comparison to healthy controls. Dietary supplementation of these vitamins increased the blood levels of these antioxidants and led to a significant reduction in depression (HAM-D)-related symptoms. Moreover, treatment with a supplement of vitamins E and C, combined with monoaminergic antidepressant drugs for 12 weeks, improved parameters of oxidative stress in MD patients (Ghodake et al., 2012). Furthermore, treatment with the antioxidant *N*-acetylcysteine improved depressive symptoms and functionality while providing good tolerability (Fernandes et al., 2016).

In sum, mitochondria represent an attractive target for drug delivery and drug development strategies because of their role in cellular energy metabolism and ROS production (Sheu et al., 2006). During the last decade, a range of strategies have been developed with the aim to target antioxidants to mitochondria. Many mitochondrially targeted antioxidants exert protective activities. Data from a series of *in vitro* systems suggest that one of them, MitoQ (a triphenylphosphonium-based mixture of ubiquinol and ubiquinone), was active in both *in vitro* and *in vivo* models of cardiac ischemia–reperfusion injury, which is associated with mitochondrial oxidative damage (Sheu et al., 2006).

Also, several studies have used antioxidant molecules such as *N*-acetylcysteine and curcumin, which target mitochondrial monoamine oxidase A, in the treatment of MD (www.clinicaltrials.gov). Among the emerging natural antioxidants for the treatment of MD, carnosine represents one of the most promising (Caruso et al., 2019b) because of its ability to counteract oxidative stress (Fresta et al., 2018; Caruso et al., 2019c), modulate energy metabolism and protect brain mitochondria (Caruso et al., 2019a), rescue mitochondrial dysfunctions (Corona et al., 2011), and interact with cells of the immune system (Fresta et al., 2017). In addition, dietary supplementation with carnosine suppressed the effects of chronic stress in animals and improved behavior, cognition, and overall well-being in human subjects (Hipkiss, 2015). As already postulated by Hipkiss some years ago, the therapeutic potential of carnosine in the treatment of stress-related and depressive disorders should be investigated intensely in future longitudinal studies.

## Conclusion and Future Directions

Overall, the data suggest an intriguing link between mitochondrial function and depression that warrants further investigation. First, mitochondria could be considered an important aid in the early diagnosis of MD and contribute to the differentiation of disorders with overlapping symptoms. Similarly, specific forms of mitochondrial dysfunction could be used as biomarkers in the evaluation of the underlying causes of the disease. Lastly, mitochondrial function may represent a promising new target for new antidepressant drugs and the development of customized therapy. Energy, behavior, and therapy constitute a triad of great scientific and health-related interest. To obtain more accurate diagnoses and more effective targeted therapies, it is necessary to invest our energy and resources to better comprehend the role of the what, why, where, and who of energy production, and therefore of mitochondria, and the mechanisms that control normal and pathological human behavior.

## Author Contributions

All authors listed have made a substantial, direct, and intellectual contribution to the work and approved it for publication.

## Conflict of Interest Statement

The authors declare that the research was conducted in the absence of any commercial or financial relationships that could be construed as a potential conflict of interest.
